# Suppression of chikungunya virus replication and differential innate responses of human peripheral blood mononuclear cells during co-infection with dengue virus

**DOI:** 10.1371/journal.pntd.0005712

**Published:** 2017-06-23

**Authors:** Mariana Ruiz Silva, José A. Aguilar Briseño, Vinit Upasani, Heidi van der Ende-Metselaar, Jolanda M. Smit, Izabela A. Rodenhuis-Zybert

**Affiliations:** Department of Medical Microbiology, University of Groningen and University Medical Center Groningen, Groningen, The Netherlands; George Mason University, UNITED STATES

## Abstract

Dengue and chikungunya are viral diseases transmitted to humans by infected *Aedes* spp. mosquitoes. With an estimated 390 million infected people per year dengue virus (DENV) currently causes the most prevalent arboviral disease. During the last decade chikungunya virus (CHIKV) has caused large outbreaks and has expanded its territory causing millions of cases in Asia, Africa and America. The viruses share a common mosquito vector and during the acute phase cause similar flu-like symptoms that can proceed to more severe or debilitating symptoms. The growing overlap in the geographical distribution of these mosquito-borne infections has led to an upsurge in reported cases of DENV/CHIKV co-infections. Unfortunately, at present we have little understanding of consequences of the co-infections to the human host. The overall aim of this study was to define viral replication dynamics and the innate immune signature involved in concurrent DENV and CHIKV infections in human peripheral blood mononuclear cells (PBMCs). We demonstrate that concomitant infection resulted in a significant reduction of CHIKV progeny and moderate enhancement of DENV production. Remarkably, the inhibitory effect of DENV on CHIKV infection occurred independently of DENV replication. Furthermore, changes in type I IFN, IL-6, IL-8, TNF-α, MCP-1 and IP-10 production were observed during concomitant infections. Notably, co-infections led to a significant increase in the levels of TNF-α and IL-6, cytokines that are widely considered to play a crucial role in the early pathogenesis of both viral diseases. In conclusion, our study reveals the interplay of DENV/CHIKV during concomitant infection and provides a framework to investigate viral interaction during co-infections.

## Author summary

Dengue virus (DENV) currently causes the most important arthropod-borne viral disease in humans. Chikungunya virus (CHIKV) re-emerged explosively in 2005–2006 afflicting millions of people in the Indian Ocean area and ever since continues its spread. The increasing co-circulation of these mosquito-borne viruses and the ability of the vector mosquitos to transmit both viruses at the same time have led to upsurge of DENV/CHIKV cases. Unfortunately, we have little understanding of their (immuno)pathogenesis. Well-balanced innate immune responses are crucial for early containment of infections with viruses, such as DENV and CHIKV. Therefore, in this study we focused on the effect of co-infection on viral replication and the innate immune responses of human peripheral blood mononuclear cells. We observed that CHIKV replication is inhibited during concomitant infection. In contrast, DENV replicated to higher titres during co-infection. Additionally, co-infections resulted in the significant increase in the production of immune-modulators that are implicated in DENV and/or CHIKV pathogenesis. Our study provides a basis for studying virus-host interactions during arbovirus co-infections and highlights the importance of investigating the innate immune response alongside the virus levels in the condition of natural co-infections.

## Introduction

Dengue virus (DENV) currently causes the most prevalent arthropod-borne human disease. Chikungunya virus (CHIKV) re-emerged in 2005–2006 with large outbreaks afflicting millions of people in the Indian Ocean areas and has spread into many (sub)tropical regions that have long been endemic for DENV. DENV and CHIKV mono-infections share many common features and cause similar acute symptoms which may lead to potentially severe (DENV) or painful chronic (CHIKV) diseases. Most of the co-infections cases are reported in South and West India, where in 2010 DENV and CHIKV co-circulated with high morbidity [1–4]. Incidence was also high in Myanmar, Sri-Lanka, Yemen, Madagascar, Nigeria and Gabon [5–10]. The currently available clinical data are insufficient to establish whether co-infections are favourable or detrimental to the host. While the majority of co-infections in India presented with symptoms very similar to those of DENV or CHIKV mono-infections, there were a few severe co-infections cases reported in Gabon and more recently in Colombia, Guatemala and Nicaragua [11–15]. The growing overlap in the geographical distribution of these two infections as well as the recent Zika outbreaks in South America, is likely to increase the prevalence and/or detection of co-infections [15,16].

Classification of DENV/CHIKV co-infection is based on either simultaneous detection of the viral RNAs or detection of DENV- and CHIKV–specific IgM antibodies in the patient’s blood [17–19]. Clearly, while the markers indicate recent dual infection, they do not discriminate whether transmission of the viruses occurred by one dually infected mosquito or a bite of two singularly infected mosquitoes. Importantly, studies of the *Aedes spp*. mosquito vectors demonstrate that a concomitant DENV and CHIKV transmission by a single mosquito is in fact, very likely [10,20–22]. The events following concomitant transmission to the human host are however unknown.

The host’s innate immune response plays an important role in the confinement and pathogenesis of DENV and CHIKV infections. In blood, DENV and CHIKV target immune cells for replication. Indeed, both viruses were shown to infect monocytes- cells that are specialized in the recognition of invading pathogens and initiation of protective immune responses [23–26]. Interestingly, viruses developed mechanisms to evade early cellular immunity of the host, for example, by antagonizing the antiviral IFN type I signalling [27–29]. In fact, viruses ensure their replication and dissemination through modulation of immune responses [30]. Importantly, the kinetics of DENV and CHIKV replication in infected cells are different. In general, the replication cycle of CHIKV is shorter, and thus it is expected to trigger and/or antagonize innate immune responses before DENV does [25,31]. As yet, little is known about the mechanisms of arboviral co-infections in humans.

In this study, we analysed virus replication kinetics and temporal changes in innate immune responses during DENV/CHIKV mono- and co-infections in human peripheral blood mononuclear cells. The co-infections were performed at distinct or identical multiplicity of infections (MOIs) since both conditions are likely to occur *in vivo* [15]. The cellular immune response was assessed by detection of pro-inflammatory cytokines and chemokines that have been implicated in the control and pathogenesis of DENV/CHIKV infection.

## Results

### Growth kinetics of CHIKV during co-infection

**Fig 1:**
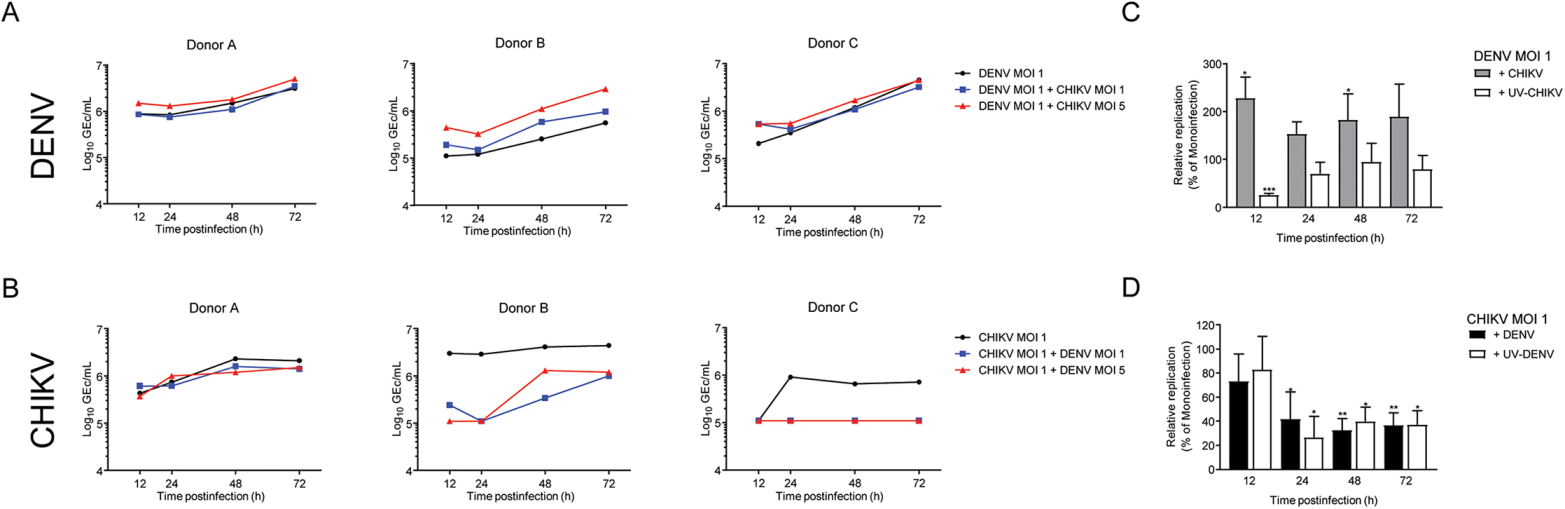
Time-course analysis of DENV and CHIKV (co)- infection in hPBMCs cell-free supernatants from PBMCs of three different donors infected with DENV or CHIKV or co-infected with both viruses were collected at 12, 24, 48 and 72hpi and analysed by qPCR to determine the number of specific viral particles expressed as genome equivalent copies per mL (GEc/mL). (A) Time-course analysis of DENV production during mono-infection at MOI of 1 and during the co-infection with CHIKV at different MOIs. (B) Time-course analysis of CHIKV production during mono-infection at MOI of 1 and during the co-infection with DENV at different MOIs. (C) Effect of co-infection on CHIKV production in hPBMCs, of DENV MOI 1 during co-infection with CHIKV or UV-CHIKV relative to mono-infection. (D) Effect of the co-infection with replicative and non-replicative DENV on CHIKV production in hPBMCs, For C and D, bars represent an average % of infection normalized to relative mono-infections of three donor’s ± SEM.

We infected PBMCs from 3 different donors with one or both viruses at various multiplicities of infection (MOIs) as described in Materials and Methods. DENV and CHIKV production was measured in the supernatants to determine the growth kinetics of the viruses during mono- and co-infection (Fig 1 panels A and B). Interestingly, despite the fact that CHIKV is usually considered to have a shorter replication cycle than DENV (approximately 12 h and 24 h, respectively), both viruses were detected in the cell supernatant as early as 12 hpi. Furthermore, in general, viral titers increased over time however CHIKV production reached a plateau between 24 and 48 hpi while DENV continued to replicate until 72 hpi. At 48 hpi, titers of both viruses were comparable between the donors, although donor B was clearly more susceptible to CHIKV than to DENV as DENV titers were 1 log lower than that of CHIKV at all harvesting times (Fig 1A vs 1B).

In case of co-infections, higher levels of DENV in the supernatants from co-infected cells were observed when compared to mono-infected cells [Fig 1A and 1C for (co)-infections at DENV MOI of 1 and S1A and S2A Figs for DENV MOI of 5]. Depending on the MOIs, the increase was statistically significant at different time points; for co-infections with DENV at MOI of 1 at 12 and 48 hpi (p<0.05) and for co-infections with DENV at MOI of 5 at 24 hpi (p<0.05) and 72 hpi (p<0.01). Interestingly, although at 12 hpi an increase in DENV replication (p<0.05) was seen during co-infection, a significant decrease (p<0.001) in replication was observed during co-infection with replication-incompetent UV-inactivated CHIKV (UV-CHIKV). At later time point, co-exposure with UV-CHIKV had no effect on DENV infection, suggesting that enhancement of DENV replication during co-infections relied on CHIKV replication. Intriguingly, when CHIKV production was assessed, we noticed a profound decrease of CHIKV titres during co-infections in all donors tested (Fig 1B and 1D for infections at CHIKV MOI of 1 and S1B and S2B Figs for CHIKV MOI of 5). The relative fold-change analysis of data from all the donors (Fig 1D) demonstrated that CHIKV production was significantly reduced from 24 hpi onwards during co-infections with DENV (p<0.05, at 24 hpi and p<0.01 at 48 and 72 hpi). To gain insight into the observed inhibition on CHIKV replication, we next compared the growth of CHIKV during co-infection with UV-DENV. Remarkably, the antagonistic effect of co-infection on CHIKV was independent of DENV replication (Fig 1D). In fact, inhibition was stronger and more consistent between the donors and different MOI conditions in presence of UV-DENV (S2D Fig).

### Mixed signature of innate immune responses during DENV/CHIKV co-infection

**Fig 2:**
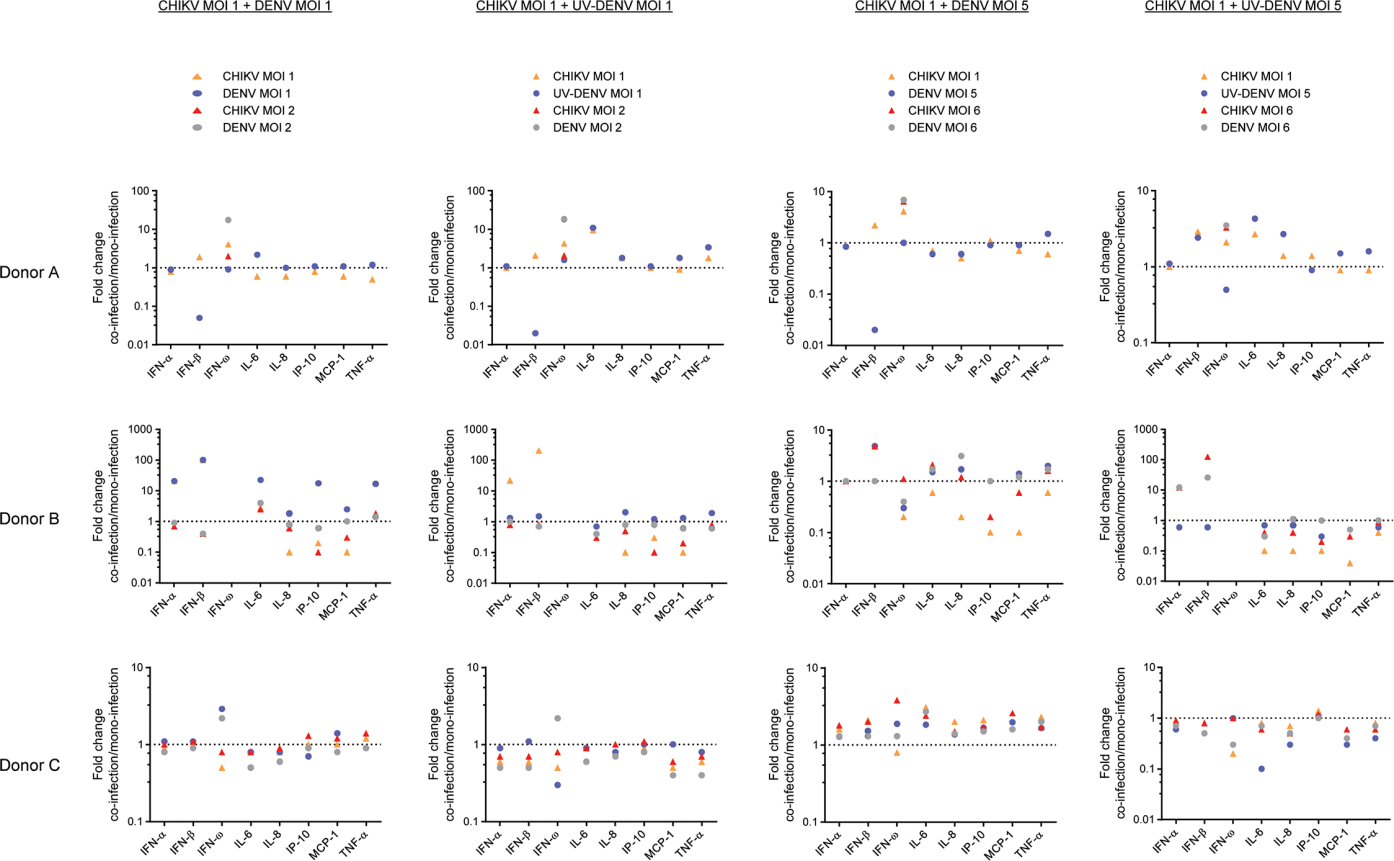
Innate immune signature of the co-infection vs mono-infection. Chemokine and cytokine levels produced following different mono- and co-infection regimens in hPBMCs were measured by ProcartaPlex. Each graph shows the fold-change of concentration of the immune factor during co-infection relative to the indicated mono-infection. All concentrations shown correspond to 24 hpi, except for that of IFN-ω, which peaked in all donors at 48 hpi.

Next, we sought to assess whether co-infection modulated the innate immune responses of the PBMCs. To this end, we selected a pool of cytokines and chemokines that are considered to play an important role in the confinement and/or pathogenesis of DENV and CHIKV infections [32–36]. We determined the concentrations of IFN-α, IFN-β, IFN-ω, IL-6, IL-8, IP-10, MCP-1 and TNF-α in the cell supernatants at 6, 24 and 48 hpi by multiplex immunoassay. As expected, the concentrations of different cytokines varied considerably between the donors, different multiplicities of infections and time points. Therefore, in Fig 2 we displayed fold-changes in the concentrations of cytokines released during co-infections relative to the corresponding mono-infection for each donor at 24 hpi, the peak time point for the vast majority of the measured cytokines (S4 Fig). When we compared fold-changes between co-infections and mono-infections within a donor, the directional trend was found to be consistent across the donors. To analyze this in more detail, we modelled log-cytokine expression values using a linear mixed model, allowing a random donor effect and a random interaction between donor and time. This model was used for all cytokines but IFN-β. For this cytokine, an additional interaction between experimental effect and time point was included due to significant, directionally opposite effects occurring between 6–24 hpi and 24–48 hpi (S4 Fig). The results of the tests are summarized in Table 1.

**Table 1:** Significant changes in cytokine levels following CHIKV and DENV mono- and co-infection in hPBMCs.

Cytokine/Chemokine	Infection	Average fold change	P value
IFN-α			
	UV-CHIKV	+1.71	P<0.001***
	UV-DENV	+1.33	P<0.05*
IFN-β^#^			
	CHIKV	+3.08	P = 0.004**
	DENV	+4.66	P<0.001***
	UV-CHIKV	+4.39	P = 0.001**
	UV-DENV	+9.53	P<0.001***
	Co-infection vs CHIKV^§^	+5.26	P = 0.003**
	Co-infection vs co-infection with UV-DENV^§^	-0.22	P = 0.025*
IFN-ω			
	CHIKV	-0.51	P = 0.004**
	Co-infection vs CHIKV	+2.80	P<0.001***
	Co-infection vs DENV	+2.28	P<0.001***
	Co-infection with UV-DENV vs CHIKV	+2.90	P<0.001***
IL-6			
	Co-infection vs DENV	+1.64	P = 0.003**
IL-8			
	Co-infection vs CHIKV	-0.64	P = 0.006**
IP-10			
	CHIKV	+1.75	P<0.001***
	UV-CHIKV	+2.09	P<0.001***
	Co-infection vs CHIKV	-0.51	P<0.001***
	Co-infection with UV-DENV vs CHIKV	-0.57	P<0.001***
MCP-1			
	CHIKV	+2.53	P<0.001***
	UV-CHIKV	+2.47	P<0.001***
	Co-infection vs CHIKV	-0.43	P<0.001***
	Co-infection with UV-DENV vs CHIKV	-0.40	P<0.001***
TNF-α			
	DENV	-0.63	P = 0.004**
	Co-infection vs CHIKV	+1.38	P = 0.011*
	Co-infection vs DENV	+1.94	P<0.001***

^#^*p* values were calculated using a linear mixed model.

^§^ as measured 24 hpi

As expected, based on the published literature [25,37,38], CHIKV mono-infection resulted in significant increase of secreted IFN-β (3.08 fold, p = 0.004), IP-10 (2.09 fold, p<0.001) and MCP-1 (2.53 fold, p<0.001) as compared to mock-infection. Interestingly, exposure of the PBMCs to UV-CHIKV also caused a significant increase in production of these immune mediators with additional stimulation of IFN-α (1.71 fold, p<0.001). The only cytokine that was found to be significantly decreased during CHIKV mono-infections was IFN-ω (0.51 fold, p = 0.004). In case of DENV mono-infections, significantly increased production of IFN-β was observed for replicative (4.66 fold, p<0.001) as well as non-replicative virus (9.53 fold, p<0.001). Exposure of the cells to UV-DENV also induced IFN-α (1.33 fold, p<0.05). Furthermore, a modest but significant inhibition of TNF-α (0.63 fold, p = 0.004) during DENV infection. None of the virus mono-infections significantly altered the levels of IL-6 and IL-8.

Immune modulators that increased during co-infections compared to CHIKV mono-infection were IFN-ω (2.8 fold, p<0.001), TNF-α (1.38 fold, p = 0.011) and IFN-β (5.26 fold, p = 0.003 at 24 hpi). IFN-ω levels were higher (2.9 fold, p<0.001) during co-infection with UV-DENV than during CHIKV mono-infection, suggesting that this IFN could have contributed to the suppression of CHIKV during co-infections. The immune modulators that were found in lower concentrations during co-infection compared to CHIKV mono-infection were IL-8 (0.64 fold, p = 0.006), IP-10 (0.51 fold, p<0.001) and MCP-1 (0.43 fold, p<0.001). These two last chemokines, IP-10 (0.57 fold, p<0.001) and MCP-1 (0.40 fold, p<0.001), also had lower concentrations during co-infections with UV-DENV than during CHIKV mono-infections. Finally, cytokines that increased during co-infection when compared to DENV mono-infection were IFN-ω (2.28 fold, p<0.001), TNF-α (1.94 fold, p<0.001) and IL-6 (1.64 fold, p = 0.003).

In order to rule out that the observed changes in the cytokine response was solely due to an increase of the total number of particles added to cells, we compared the fold changes triggered by co-infections vs mono-infections with the same total number of particles (Table 2). Thus, the mono-infections at MOI of 2, 6, 10 were used as controls of the effect of viral particle number on innate responses during co-infections (1:1), (1:5)/(5:1), (5:5), respectively. Importantly, the majority of the cytokines that were significantly altered during co-infection as presented in Fig 2 and summarized in Table 1 did not withstand the particle control analysis. Only IFN-ω, TNF-α, IL-6 and IP-10 were in fact differentially modulated during co-infections. The levels of IFN-ω during co-infection were significantly higher than those of the mono-infections with DENV (3.34 fold, p<0.001) and CHIKV (2.83 fold, p<0.001) with comparable MOIs. The same pattern was found for TNF-α, levels of which were significantly increased in co-infection with 1.86 fold (p = 0.007) and 2.16 fold (p = 0.001) as compared to CHIKV and DENV mono-infections, respectively. Interestingly, the significance in differential modulation of IP-10 and IL-6 depended on which virus mono-infection they were compared to. The level of IP-10 was lower during co-infection (0.66 fold, p = 0.0244) than during particle-matched mono-infection of CHIKV. Yet, when compared to the same amount of DENV particles, there was no significant difference in the IP-10 levels between co- and mono-infections. This implies that co-infection altered the relative IP-10 response to CHIKV but not to DENV. In contrast, IL-6 was upregulated during co-infection when compared to DENV particle-matched mono-infection (1.72 fold, p = 0.047) but not to that of CHIKV. In summary, our data strongly indicate that co-infection modulates the innate immune response of the respective mono-infections.

**Table 2:** Changes in cytokine signature: Co-infection vs particle control.

	Co-infections vs DENV particle controls^#^		Co-infections vs CHIKV particle controls^#^	
Cytokine/ chemokine	Average fold change	P value	Average fold change	P value
IFN-α	+1.21	0.243	+1.19	0.283
IFN-β^@^	-0.64	0.613	+2.25	0.343
IFN-ω	**+3.34**	<0.001***	**+2.83**	<0.001***
IL-6	**+1.72**	0.047 *	+1.39	0.215
IL-8	+1.70	0.121	+1.23	0.533
IP-10	+1.38	0.086	**-0.66**	0.024**
MCP-1	+1.37	0.348	-0.78	0.442
TNF-α	**+2.16**	0.001**	**+1.86**	0.007**

^#^ p values were calculated using a linear mixed model as described in the Methods section

^@^ values for 24hpi

### Type I IFN response is different in co-infection vs mono-infection and potentially contributes to decreased CHIKV replication during co-infection

**Fig 3:**
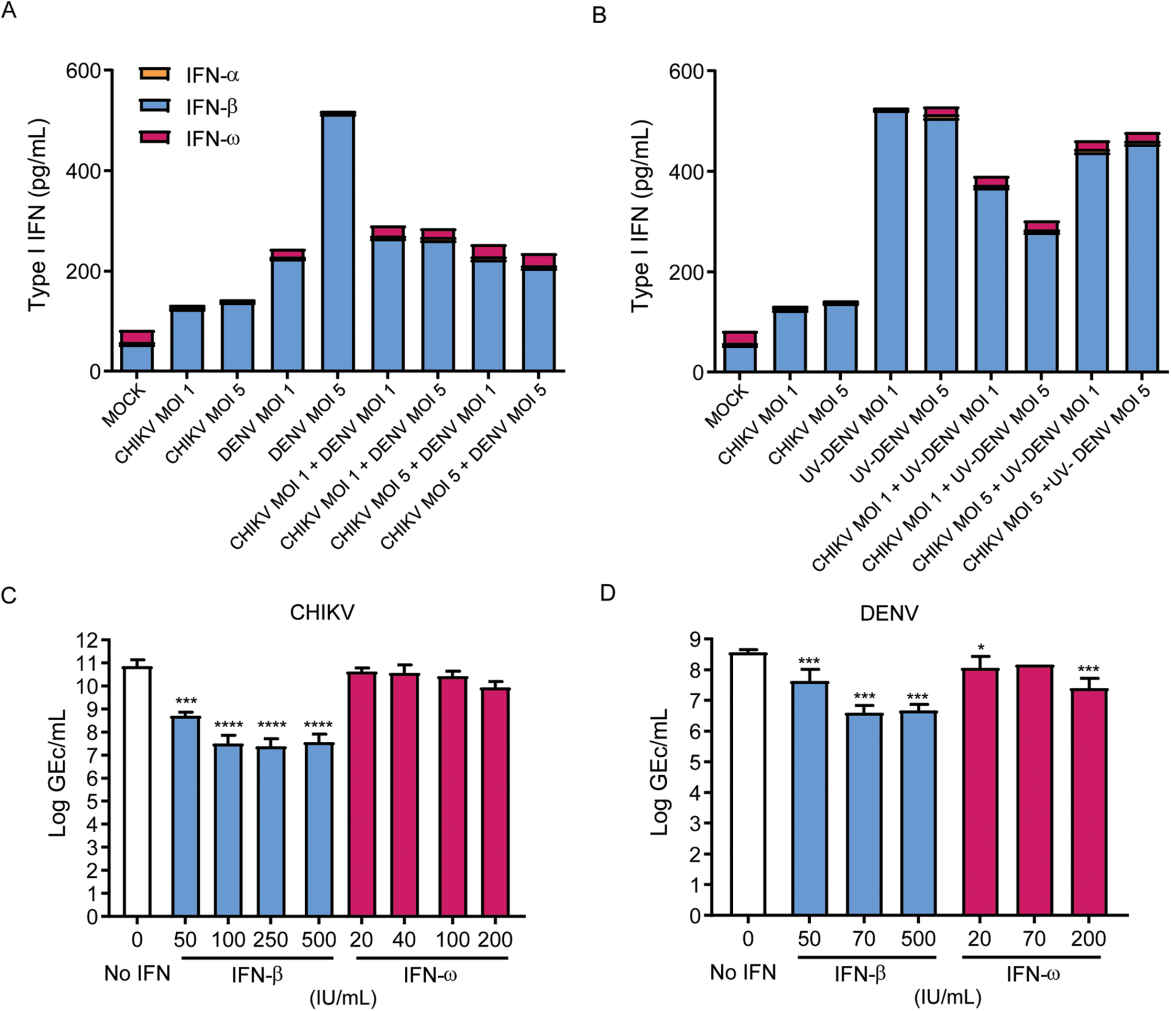
Differential role of type I interferons in CHIKV and DENV infection. (A) Concentration of IFN-α, IFN-β and IFN-ω in the supernatants of mono- and co-infected cells at 24 hpi. (B) Concentration of IFN-α, IFN-β and IFN-ω in the supernatants of mono- and CHIKV/UV-DENV co-infected cells at 24 hpi. (C/D) Effect of IFN-β and IFN-ω on CHIKV and DENV replication. Vero-WHO cells were pre-treated with IFN-β or IFN-ω for 24 h and infected with CHIKV (C) or DENV (D) at MOI of 1. The number of genome equivalent copies per mL (GEc/mL) in cell-free supernatant harvested at 24 hpi was determined by RT-qPCR. Results are represented as mean ± SEM of three independent experiments.

Lastly, we sought to test whether suppression of CHIKV infections observed in our experiments could be attributed to differential induction of type I interferons during co-infection (in Table 1). Type I interferons are part of the first line of defense against many viral infections and their rapid induction can limit infection and viral spread [39]. Since inhibition of CHIKV replication during co-infection occurred independently of DENV replication (Fig 1D), we reasoned that co-infections, in particular those with non-replicating UV-DENV, led to higher levels of one or more antiviral interferons that ultimately inhibited CHIKV infection. Therefore, we re-analysed the data to depict changes in the concentrations of individual IFNs (IFN-α, IFN-β and IFN-ω) as well as the collective IFN type I response in co- vs mono-infection (Fig 3A). Indeed, the cumulative level of type I IFN was clearly higher in co-infections than in mono-infections with CHIKV. Since IFN-β and IFN-ω were the most prevalent cytokines in supernatants from mono- and co-infections, we next focussed on their role as potential antiviral effectors. To the best of our knowledge, the antiviral effect of IFN-ω on CHIKV infection has never been evaluated. To assess the antiviral activity of interferons on the individual viruses we used an assay based on IFN-deficient Vero-WHO cells [40]. Briefly, cells were pre-treated with mock- or increasing concentrations of IFN-β or IFN-ω for 24 h and subsequently infected with CHIKV or DENV at various MOIs (Fig 3B and 3C respectively). Interestingly, IFN-ω had no effect on CHIKV infection whereas it significantly inhibited (p = 0.011) DENV infection already at 20 IU/mL (Fig 3B). Hence, IFN-ω might have contributed the transient but significant reduction/delay of DENV production observed at 12 hpi (Fig 1C). As expected [40,41], IFN-β pre-treatment inhibited infection of both viruses. Notably, however, IFN-β pre-treatment had a much stronger effect on CHIKV than on DENV (50-fold vs 8-fold infection inhibition, respectively). Consequently, the differential susceptibility to IFN-β might explain why increased production of IFN-β during co-infection selectively inhibited CHIKV but not DENV infection.

## Discussion

Minimal research has been performed investigating the immune responses and cellular mechanisms of arboviral co-infections [42]. The majority of the studies are performed in *Aedes* mosquitoes or cell lines and focus on the transmission [21,22,43]. To our knowledge, this is the first *in vitro* study describing the effects of DENV and CHIKV co-infection in human cells.

We found that simultaneous co-infections result in the selective inhibition of CHIKV replication and an increase in cytokines associated with disease severity such as TNF-α and IL-6. Interestingly, similar observations were made by Waggoner et al., who reported lower viremia in DENV/CHIKV co-infected patients than those infected with one virus, yet the co-infected patients required more frequent hospitalization than the mono-infected ones [15]. However, the direct association of our findings to those gathered in clinical study should be done with caution as in natural co-infections it is uncertain whether they occurred simultaneously or sequentially. Indeed, Caron et al. described two groups of co-infected patients, one with high DENV-2 titers and low CHIKV titers, and a second group with high titers of both viruses [10]. In light of our results, it is tempting to speculate that the first group included patients with simultaneous co-infections, while the second those who acquired the viruses consecutively. However, since our study did not address the sequential co-infections, we can only coincide with the postulates of Caron et al., that whether the viruses were transmitted in a short time frame (either by the same mosquito or two mosquitos) or with days apart may be defining for the host immune response and consequently the observed phenotype. Regardless, all these observations highlight the importance of investigating the innate immune response next to the viremia levels in the condition of natural co-infections.

Downregulation of CHIKV production during co-infection with DENV was independent of the ability of the latter virus to establish infection. This suggests that incoming dengue virions triggers innate immune receptor signalling to convey CHIKV-antagonistic responses. Several pathogen recognition receptors (PRRs) have been implicated in the sensing of DENV upon entry including TLR7/8, CLEC5, DCIR [44,45]. We are currently investigating, which of the PRRs contribute to the initiation of CHIKV-antagonistic responses.

The mixed signature of immune-modulators found in this study corroborates the similarities between the innate responses triggered by these viruses. Although levels of TNF-α and IL-6 following mono-infections were donor and MOI-dependent; production of these cytokines was significantly augmented during co-infections and this was not attributed to the increase in total MOI. This finding deserves further investigation since these cytokines are considered to be innate predictors of severe dengue [46] and severe chikungunya disease [47]. Interestingly, several immune mediators such as IFN-ω, IP-10 and MCP-1 were differentially expressed when the cells were exposed simultaneously to both viruses. For instance, we observed suppression of IFN-ω in PBMCs following both CHIKV and DENV mono-infections, however the level of this interferon during co-infections was higher than during mono-infection. The mechanism behind the differential expression of IFN-ω during co-infection is a subject of interest for future investigation in particular due to the different susceptibility of the viruses to type I IFNs. Also, IP-10 and MCP-1 production was stimulated upon infection with CHIKV and reduced during co-infection. Although this may indicate that these chemokines are important in CHIKV replication we have recently shown that MCP-1 does not play a role in CHIKV replication [38]. The effect of IP-10 on CHIKV replication is unknown however it has been found to increase the replication of human immunodeficiency virus 1 (HIV-1) by increasing the accumulation of HIV-1 DNA in infected cells [48].

To our knowledge this is the first systematic study that addresses the viral kinetics and innate immune responses upon DENV and CHIKV co-infection performed in primary human cells. Importantly, the use of particle controls was essential to rule out an effect observed solely by the total increase of number of virions in co-infections and not by the interplay of two different viruses. Yet, our study does not address how sequential co-infections impact the host’s response and viral production. This as well as the effect of co-infections on the adaptive immunity will be a subject of our forthcoming investigations. Altogether, the unexpected downregulation of CHIKV and altered immune signature during co-infections show the complicated nature of the interplay of the viruses during co-infections. Due to the re-emergence of CHIKV with higher morbidity and rapidly expanding co-circulation of CHIKV, DENV and now also Zika virus we are facing an upsurge of arboviral co-infection cases.

## Methods

### Cells

Vero E6 (a gift from dr. G. Pijlman, Wageningen University) and Vero WHO (ATCC) were cultured in DMEM (Life Technologies) containing 10% Fetal Bovine Serum (FBS), penicillin (100 U/ml), streptomycin (100 μg/ml), 10 mM HEPES, and 200 mM glutamine. PBMCs were maintained in RPMI 1640 medium supplemented with 10% FBS. Human PBMCs were isolated from Buffy coats using standard density gradient centrifugation procedures with Ficoll-Paque PLUS (GE Healthcare), as described previously. The buffy coats were obtained from healthy volunteers with informed consent from Sanquin blood bank, in line with the declaration of Helsinki. The PBMCs were cryopreserved at -196°C.

### Viruses and virus titrations

CHIKV (La Reunion OPY1) was a gift from A. Merits (University of Tartu, Estonia), and was produced from infectious cDNA clones and passaged twice in Vero E6 cells [49]. DENV-2 strain 16681 was propagated in *Aedes albopictus* cell line C6/36, as described before [50]. Both virus preparations were analysed with respect to the infectious titre and the number of genome equivalents copies as described previously. Briefly, the infectivity of DENV was determined by measuring the number of plaque-forming units (PFU) by plaque assay on BHK-15 cells and the number of genome-equivalent copies (GEc) by quantitative RT-PCR (qRT-PCR), as described previously [51,52]. For CHIKV, the infectious virus titer was determined by standard plaque assay on Vero-WHO cells at 37°C and reverse transcriptase quantitative PCR (RT-qPCR) was used to determine the number of genome equivalents copies (GEc) [53].

### UV inactivation

Virus inactivation was obtained by 1.5 h incubation of virus aliquots under UVS-28 8 watt Lamp. Inactivation to below level of detection 35 PFU/mL was assessed using standard plaque assay in Vero-WHO cells or BHK-15 as described previously, for CHIKV or DENV, respectively [51,53].

### Infection of PBMCs

PBMCs from three different donors were infected with DENV and/or CHIKV multiplicity of infection (MOI) 1 or 5 at 37°C as described previously for mono-infections [38]. Cells were also exposed to UV-inactivated CHIKV and DENV in single exposures or in combination with the other virus replicative form. The mono-infections at MOI of 2, 6, 10 were used as controls of the effect of viral particle number. After 2 h incubation at 37°C, the inoculum was removed and fresh medium was added to the cells. At each indicated time point, cell-free supernatant was collected, divided into 2 aliquots and stored for subsequent analyses of cytokine and virus production.

### Cytokines and chemokines determinations

Concentrations of IFN-α, IFN-β, IFN-ω, IL-6, IL-8, MCP-1, IP-10, and TNF-α were measured in cell-free supernatants using ProcartaPlex and human IFN-ω platinum ELISA (both from eBioscience) according to respective manufacturer’s instructions.

### Effect of type I IFNs on CHIKV and DENV replication

Vero WHO cells (2x10^5^ cells/well) were (mock)treated with various concentrations of IFN-β (50, 70, 100, 250 and 500 IU/mL) or IFN-ω (20, 40, 100 and 200 IU/mL) (PROSPEC, cat #: CYT-040) at 37°C. After 24 h cells were washed twice with DMEM and (mock)infected with CHIKV-LR or DENV-2 at the MOI of 1at 37°C. At 2 hpi the inoculum was removed, cells were washed once with DMEM and finally resuspended in DMEM supplemented with 5% FBS. Cell-free supernatants were collected after 24 h of incubation at 37°C and stored for subsequent analysis of virus production by qPCR.

### Statistics

All data are expressed as mean with bars representing standard error of the mean (SEM), unless specified otherwise. Linear mixed models were estimated using R package ‘lme4’ and contrasts tested with ‘multcomp’. Changes in viral titers were analysed using Student’s *t-*test in GraphPad Prism 5 application. In all tests, values of *p<0.05, **p<0.01 and ***p<0.001 were considered significant.

## Supporting information

S1 Fig Time-course analysis of DENV and CHIKV (co)- infection in hPBMCs. (A) Kinetics of DENV MOI 5 during mono-infection or co-infection with CHIKV and UV-CHIKV at different MOIs. (B) Kinetics of CHIKV MOI 5 during mono-infection or during co-infection with DENV and UV-DENV at different MOIs. (C) Kinetics of DENV MOI 1 during mono-infection or during co-infection with UV-CHIKV. (D) Kinetics of CHIKV MOI 1 during mono-infection and during co-infection with UV-DENV.
(TIF)

S2 Fig Effect of co-infection on CHIKV or DENV production in hPBMCs. (A) Production of DENV MOI 5 during co-infection with CHIKV or UV-CHIKV relative to mono-infection; (B) Replication of CHIKV MOI 5 during co-infection with DENV or UV-DENV relative to CHIKV mono-infection; (C) Combined effects of co-infection (all MOIs) on DENV mono-infection; (D) Combined effects of co-infections on CHIKV mono-infections. Results are represented as mean ± SEM of three donors.
(TIF)

S3 Fig Innate immune signature of co-infection vs mono-infection. Each graph shows the fold-change of concentration of the immune factor during co-infection relative to the indicated mono-infection. All concentrations shown correspond to 24 hpi, except for that of IFN-ω, which was detectable on all donors at 48 hpi.
(TIF)

S4 Fig Time-course analysis of cytokines and chemokines production following CHIKV and DENV mono- or co-infection. Each graph shows kinetics of the immune factor production during mono- and co-infections. High/ low represents results for MOI 1, 2 and MOI 5, 6 and MOI 10 respectively. All available data of 3 donors are shown.
(PDF)

## References

[1] Chahar HS, Bharaj P, Dar L, Guleria R, Kabra SK, Broor S. Co-infections with chikungunya virus and dengue virus in Delhi, India. Emerging Infect Dis 2009;15(7):1077–1080. doi: 10.3201/eid1507.080638 19624923 10.3201/eid1507.080638 PMC2744227

[2] Singh P, Mittal V, Rizvi M, Chhabra M, Sharma P, Rawat D, et al The first dominant co-circulation of both dengue and chikungunya viruses during the post-monsoon period of 2010 in Delhi, India. Epidemiol Infect 2012;140(07):1337–1342. 21906409 10.1017/S0950268811001671

[3] Taraphdar D, Sarkar A, Mukhopadhyay BB, Chatterjee S. A comparative study of clinical features between monotypic and dual infection cases with Chikungunya virus and dengue virus in West Bengal, India. Am J Trop Med Hyg 2012 4;86(4):720–723. doi: 10.4269/ajtmh.2012.11-0704 22492160 10.4269/ajtmh.2012.11-0704 PMC3403770

[4] Afreen N, Deeba F, Khan WH, Haider SH, Kazim SN, Ishrat R, et al Molecular characterization of dengue and chikungunya virus strains circulating in New Delhi, India. Microbiol Immunol 2014;58(12):688–696. doi: 10.1111/1348-0421.12209 25346397 10.1111/1348-0421.12209

[5] Tun MM, Thant KZ, Inoue S, Nabeshima T, Aoki K, Kyaw AK, et al Detection of east/central/south African genotype of chikungunya virus in Myanmar, 2010. Emerg Infect Dis 2014 8;20(8):1378–1381. doi: 10.3201/eid2008.131431 25062511 10.3201/eid2008.131431 PMC4111191

[6] Kularatne SA, Gihan MC, Weerasinghe SC, Gunasena S. Concurrent outbreaks of Chikungunya and Dengue fever in Kandy, Sri Lanka, 2006–07: a comparative analysis of clinical and laboratory features. Postgrad Med J 2009 7;85(1005):342–346. doi: 10.1136/pgmj.2007.066746 19581242 10.1136/pgmj.2007.066746

[7] Rezza G, El-Sawaf G, Faggioni G, Vescio F, Al Ameri R, De Santis R, et al Co-circulation of Dengue and Chikungunya Viruses, Al Hudaydah, Yemen, 2012. Emerg Infect Dis 2014 8;20(8):1351–1354. doi: 10.3201/eid2008.131615 25061762 10.3201/eid2008.131615 PMC4111199

[8] Ratsitorahina M, Harisoa J, Ratovonjato J, Biacabe S, Reynes J, Zeller H, et al Outbreak of dengue and Chikungunya fevers, Toamasina, Madagascar, 2006. Emerging Infect Dis 2008;14(7):1135–1137. doi: 10.3201/eid1407.071521 18598641 10.3201/eid1407.071521 PMC2600361

[9] Baba M, Logue CH, Oderinde B, Abdulmaleek H, Williams J, Lewis J, et al Evidence of arbovirus co-infection in suspected febrile malaria and typhoid patients in Nigeria. The Journal of Infection in Developing Countries 2013;7(01):051–059. 10.3855/jidc.2411 23324821

[10] Caron M, Paupy C, Grard G, Becquart P, Mombo I, Nso BB, et al Recent introduction and rapid dissemination of Chikungunya virus and Dengue virus serotype 2 associated with human and mosquito coinfections in Gabon, central Africa. Clin Infect Dis 2012 9;55(6):e45–53. doi: 10.1093/cid/cis530 22670036 10.1093/cid/cis530

[11] Gandhi BS, Kulkarni K, Godbole M, Dole SS, Kapur S, Satpathy P, et al Dengue and Chikungunya coinfection associated with more severe clinical disease than mono-infection. Int J Healthcare Biomed Res 2015;3(3):117–123.

[12] Leroy EM, Nkoghe Mba D, Ollomo B, Nze-Nkogue C, Becquart P, Grard G, et al Concurrent chikungunya and dengue virus infections during simultaneous outbreaks, Gabon, 2007. Emerging infectious diseases 2009;15(4):591–593. doi: 10.3201/eid1504.080664 19331740 10.3201/eid1504.080664 PMC2671412

[13] Mercado M, Acosta-Reyes J, Parra E, Pardo L, Rico A, Campo A, et al Clinical and histopathological features of fatal cases with dengue and chikungunya virus co-infection in Colombia, 2014 to 2015. Euro Surveill 2016 6 2;21(22): doi: 10.2807/1560-7917.ES.2016.21.22.30244 27277216 10.2807/1560-7917.ES.2016.21.22.30244

[14] Edwards T, Signor LD, Williams C, Donis E, Cuevas LE, Adams ER. Co-infections with Chikungunya and Dengue Viruses, Guatemala, 2015. Emerg Infect Dis 2016 11;22(11):2003–2005. doi: 10.3201/eid2211.161017 27767914 10.3201/eid2211.161017 PMC5088021

[15] Waggoner JJ, Gresh L, Vargas MJ, Ballesteros G, Tellez Y, Soda KJ, et al Viremia and Clinical Presentation in Nicaraguan Patients Infected with Zika Virus, Chikungunya Virus, and Dengue Virus. Clin Infect Dis 2016 8 30. 10.1093/cid/ciw589 PMC5146717 27578819

[16] Villamil-Gómez WE, González-Camargo O, Rodriguez-Ayubi J, Zapata-Serpa D, Rodriguez-Morales AJ. Dengue, chikungunya and Zika co-infection in a patient from Colombia. Journal of infection and public health 2016. 10.1016/j.jiph.2015.12.002 26754201

[17] Cabral-Castro MJ, Cavalcanti MG, Peralta RHS, Peralta JM. Molecular and serological techniques to detect co-circulation of DENV, ZIKV and CHIKV in suspected dengue-like syndrome patients. Journal of Clinical Virology 2016;82:108–111. doi: 10.1016/j.jcv.2016.07.017 27479173 10.1016/j.jcv.2016.07.017

[18] Pabbaraju K, Wong S, Gill K, Fonseca K, Tipples GA, Tellier R. Simultaneous detection of Zika, Chikungunya and Dengue viruses by a multiplex real-time RT-PCR assay. Journal of Clinical Virology 2016;83:66–71. doi: 10.1016/j.jcv.2016.09.001 27614319 10.1016/j.jcv.2016.09.001

[19] Waggoner JJ, Ballesteros G, Gresh L, Mohamed-Hadley A, Tellez Y, Sahoo MK, et al Clinical evaluation of a single-reaction real-time RT-PCR for pan-dengue and chikungunya virus detection. Journal of Clinical Virology 2016;78:57–61. doi: 10.1016/j.jcv.2016.01.007 26991052 10.1016/j.jcv.2016.01.007 PMC4836994

[20] Vazeille M, Mousson L, Martin E, Failloux A. Orally co-infected Aedes albopictus from La Reunion Island, Indian Ocean, can deliver both dengue and chikungunya infectious viral particles in their saliva. PLoS Negl Trop Dis 2010;4(6):e706 doi: 10.1371/journal.pntd.0000706 20544013 10.1371/journal.pntd.0000706 PMC2882319

[21] Potiwat R, Komalamisra N, Thavara U, Tawatsin A, Siriyasatien P. Competitive suppression between chikungunya and dengue virus in Aedes albopictus c6/36 cell line. Southeast Asian J Trop Med Public Health 2011;42(6):1388 22299407

[22] Ruckert C, Weger-Lucarelli J, Garcia-Luna SM, Young MC, Byas AD, Murrieta RA, et al Impact of simultaneous exposure to arboviruses on infection and transmission by Aedes aegypti mosquitoes. Nat Commun 2017 5 19;8:15412 doi: 10.1038/ncomms15412 28524874 10.1038/ncomms15412 PMC5454532

[23] Kou Z, Quinn M, Chen H, Rodrigo W, Rose RC, Schlesinger JJ, et al Monocytes, but not T or B cells, are the principal target cells for dengue virus (DV) infection among human peripheral blood mononuclear cells. J Med Virol 2008;80(1):134–146. doi: 10.1002/jmv.21051 18041019 10.1002/jmv.21051

[24] Chen YC, Wang SY. Activation of terminally differentiated human monocytes/macrophages by dengue virus: productive infection, hierarchical production of innate cytokines and chemokines, and the synergistic effect of lipopolysaccharide. J Virol 2002 10;76(19):9877–9887. doi: 10.1128/JVI.76.19.9877-9887.2002 12208965 10.1128/JVI.76.19.9877-9887.2002 PMC136495

[25] Her Z, Malleret B, Chan M, Ong EK, Wong SC, Kwek DJ, et al Active infection of human blood monocytes by Chikungunya virus triggers an innate immune response. J Immunol 2010 5 15;184(10):5903–5913. doi: 10.4049/jimmunol.0904181 20404274 10.4049/jimmunol.0904181

[26] Labadie K, Larcher T, Joubert C, Mannioui A, Delache B, Brochard P, et al Chikungunya disease in nonhuman primates involves long-term viral persistence in macrophages. J Clin Invest 2010 3;120(3):894–906. doi: 10.1172/JCI40104 20179353 10.1172/JCI40104 PMC2827953

[27] Fros JJ, Liu WJ, Prow NA, Geertsema C, Ligtenberg M, Vanlandingham DL, et al Chikungunya virus nonstructural protein 2 inhibits type I/II interferon-stimulated JAK-STAT signaling. J Virol 2010 10;84(20):10877–10887. doi: 10.1128/JVI.00949-10 20686047 10.1128/JVI.00949-10 PMC2950581

[28] Castillo Ramirez JA, Urcuqui-Inchima S. Dengue virus control of type I IFN responses: A history of manipulation and control. Journal of Interferon & Cytokine Research 2015;35(6):421–430. 25629430 10.1089/jir.2014.0129 PMC4490770

[29] Wuerth JD, Weber F. Phleboviruses and the type I interferon response. Viruses 2016;8(6):174. 10.3390/v8060174 PMC4926194 27338447

[30] Bowie AG, Unterholzner L. Viral evasion and subversion of pattern-recognition receptor signalling. Nature Reviews Immunology 2008;8(12):911–922. doi: 10.1038/nri2436 18989317 10.1038/nri2436 PMC7097711

[31] Lindenbach BD, Rice C. Flaviviridae: the viruses and their replication. Fields virology 2001;1:991–1041.

[32] Kelvin AA, Banner D, Silvi G, Moro ML, Spataro N, Gaibani P, et al Inflammatory cytokine expression is associated with chikungunya virus resolution and symptom severity. PLoS Negl Trop Dis 2011;5(8):e1279 doi: 10.1371/journal.pntd.0001279 21858242 10.1371/journal.pntd.0001279 PMC3156690

[33] Reddy V, Mani RS, Desai A, Ravi V. Correlation of plasma viral loads and presence of Chikungunya IgM antibodies with cytokine/chemokine levels during acute Chikungunya virus infection. J Med Virol 2014;86(8):1393–1401. doi: 10.1002/jmv.23875 24523146 10.1002/jmv.23875

[34] Wauquier N, Becquart P, Nkoghe D, Padilla C, Ndjoyi-Mbiguino A, Leroy EM. The acute phase of Chikungunya virus infection in humans is associated with strong innate immunity and T CD8 cell activation. J Infect Dis 2011 7 1;204(1):115–123. doi: 10.1093/infdis/jiq006 21628665 10.1093/infdis/jiq006 PMC3307152

[35] Costa VV, Fagundes CT, Souza DG, Teixeira MM. Inflammatory and innate immune responses in dengue infection: protection versus disease induction. The American journal of pathology 2013;182(6):1950–1961. doi: 10.1016/j.ajpath.2013.02.027 23567637 10.1016/j.ajpath.2013.02.027

[36] Arias J, Valero N, Mosquera J, Montiel M, Reyes E, Larreal Y, et al Increased expression of cytokines, soluble cytokine receptors, soluble apoptosis ligand and apoptosis in dengue. Virology 2014;452:42–51. doi: 10.1016/j.virol.2013.12.027 24606681 10.1016/j.virol.2013.12.027

[37] Schilte C, Couderc T, Chretien F, Sourisseau M, Gangneux N, Guivel-Benhassine F, et al Type I IFN controls chikungunya virus via its action on nonhematopoietic cells. J Exp Med 2010 2 15;207(2):429–442. doi: 10.1084/jem.20090851 20123960 10.1084/jem.20090851 PMC2822618

[38] Ruiz Silva M, van der Ende-Metselaar H, Mulder HL, Smit JM, Rodenhuis-Zybert IA. Mechanism and role of MCP-1 upregulation upon chikungunya virus infection in human peripheral blood mononuclear cells. Sci Rep 2016 8 25;6:32288 doi: 10.1038/srep32288 27558873 10.1038/srep32288 PMC4997611

[39] McNab F, Mayer-Barber K, Sher A, Wack A, O'garra A. Type I interferons in infectious disease. Nature Reviews Immunology 2015;15(2):87–103. doi: 10.1038/nri3787 25614319 10.1038/nri3787 PMC7162685

[40] Diamond MS, Roberts TG, Edgil D, Lu B, Ernst J, Harris E. Modulation of Dengue virus infection in human cells by alpha, beta, and gamma interferons. J Virol 2000 6;74(11):4957–4966. 10799569 10.1128/jvi.74.11.4957-4966.2000 PMC110847

[41] Sourisseau M, Schilte C, Casartelli N, Trouillet C, Guivel-Benhassine F, Rudnicka D, et al Characterization of reemerging chikungunya virus. PLoS Pathog 2007 6;3(6):e89 doi: 10.1371/journal.ppat.0030089 17604450 10.1371/journal.ppat.0030089 PMC1904475

[42] Deeba F, Afreen N, Islam A, Naqvi IH, Broor S, Ahmed A, et al Co-infection with Dengue and Chikungunya Viruses. Current Topics in Chikungunya 2016.

[43] Göertz GP, Vogels CB, Geertsema C, Koenraadt CJ, Pijlman GP. Mosquito co-infection with Zika and chikungunya virus allows simultaneous transmission without affecting vector competence of Aedes aegypti. PLoS Neglected Tropical Diseases 2017;11(6). 10.1371/journal.pntd.0005654 PMC5469501 28570693

[44] Wang JP, Liu P, Latz E, Golenbock DT, Finberg RW, Libraty DH. Flavivirus activation of plasmacytoid dendritic cells delineates key elements of TLR7 signaling beyond endosomal recognition. J Immunol 2006 11 15;177(10):7114–7121. 17082628 10.4049/jimmunol.177.10.7114

[45] Chen S, Lin Y, Huang M, Wu M, Cheng S, Lei H, et al CLEC5A is critical for dengue-virus-induced lethal disease. Nature 2008;453(7195):672–676. doi: 10.1038/nature07013 18496526 10.1038/nature07013

[46] John DV, Lin Y, Perng GC. Biomarkers of severe dengue disease–a review. J Biomed Sci 2015;22(1):83. 26462910 10.1186/s12929-015-0191-6 PMC4604634

[47] Ng LF, Chow A, Sun Y, Kwek DJ, Lim P, Dimatatac F, et al IL-1β, IL-6, and RANTES as biomarkers of Chikungunya severity. PloS one 2009;4(1):e4261 doi: 10.1371/journal.pone.0004261 19156204 10.1371/journal.pone.0004261 PMC2625438

[48] Lane BR, King SR, Bock PJ, Strieter RM, Coffey MJ, Markovitz DM. The CXC chemokine IP-10 stimulates HIV-1 replication. Virology 2003;307(1):122–134. 12667820 10.1016/s0042-6822(02)00045-4

[49] Pohjala L, Utt A, Varjak M, Lulla A, Merits A, Ahola T, et al Inhibitors of alphavirus entry and replication identified with a stable Chikungunya replicon cell line and virus-based assays. PLoS One 2011;6(12):e28923 doi: 10.1371/journal.pone.0028923 22205980 10.1371/journal.pone.0028923 PMC3242765

[50] Rodenhuis-Zybert IA, van der Schaar HM, da Silva Voorham JM, van der Ende-Metselaar H, Lei HY, Wilschut J, et al Immature dengue virus: a veiled pathogen? PLoS Pathog 2010 1;6(1):e1000718 doi: 10.1371/journal.ppat.1000718 20062797 10.1371/journal.ppat.1000718 PMC2798752

[51] van der Schaar HM, Rust MJ, Waarts BL, van der Ende-Metselaar H, Kuhn RJ, Wilschut J, et al Characterization of the early events in dengue virus cell entry by biochemical assays and single-virus tracking. J Virol 2007 11;81(21):12019–12028. doi: 10.1128/JVI.00300-07 17728239 10.1128/JVI.00300-07 PMC2168764

[52] Zybert IA, van der Ende-Metselaar H, Wilschut J, Smit JM. Functional importance of dengue virus maturation: infectious properties of immature virions. J Gen Virol 2008 12;89(Pt 12):3047–3051. doi: 10.1099/vir.0.2008/002535-0 19008392 10.1099/vir.0.2008/002535-0

[53] van Duijl-Richter MK, Blijleven JS, van Oijen AM, Smit JM. Chikungunya virus fusion properties elucidated by single-particle and bulk approaches. J Gen Virol 2015;96(8):2122–2132. doi: 10.1099/vir.0.000144 25872739 10.1099/vir.0.000144

