# Supplementary figures and images for: Suppression of chikungunya virus replication and differential innate responses of human peripheral blood mononuclear cells during co-infection with dengue virus
Source: PLoS Negl Trop Dis. 2017 Jun 23;11(6):e0005712. doi: 10.1371/journal.pntd.0005712 (PMC5500378; doi:10.1371/journal.pntd.0005712)

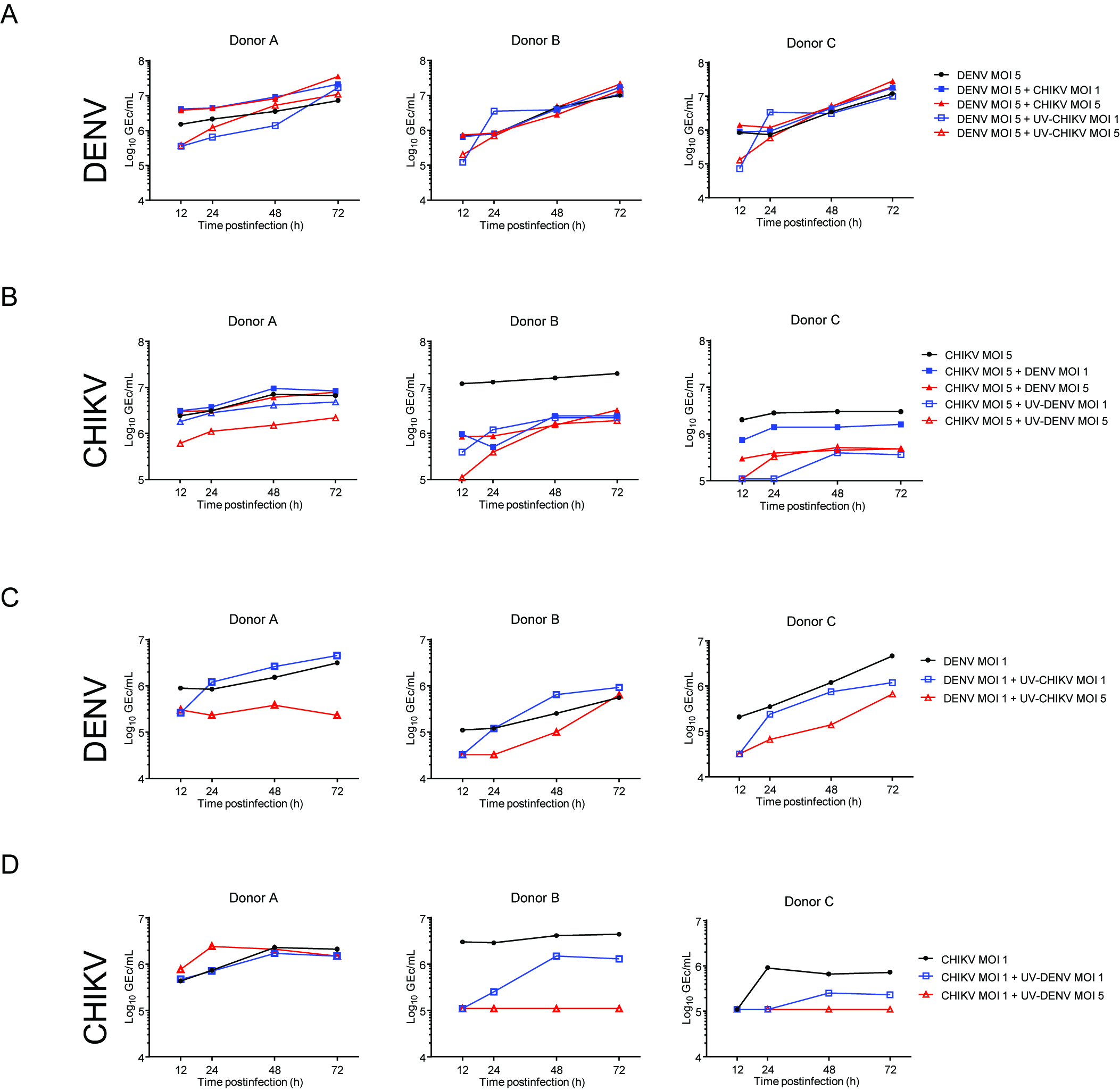

Supplement: S1 Fig — (A) Kinetics of DENV MOI 5 during mono-infection or co-infection with CHIKV and UV-CHIKV at different MOIs. (B) Kinetics of CHIKV MOI 5 during mono-infection or during co-infection with DENV and UV-DENV at different MOIs. (C) Kinetics of DENV MOI 1 during mono-infection or during co-infection with UV-CHIKV. (D) Kinetics of CHIKV MOI 1 during mono-infection and during co-infection with UV-DENV. (TIF) [file pntd.0005712.s001.tif]

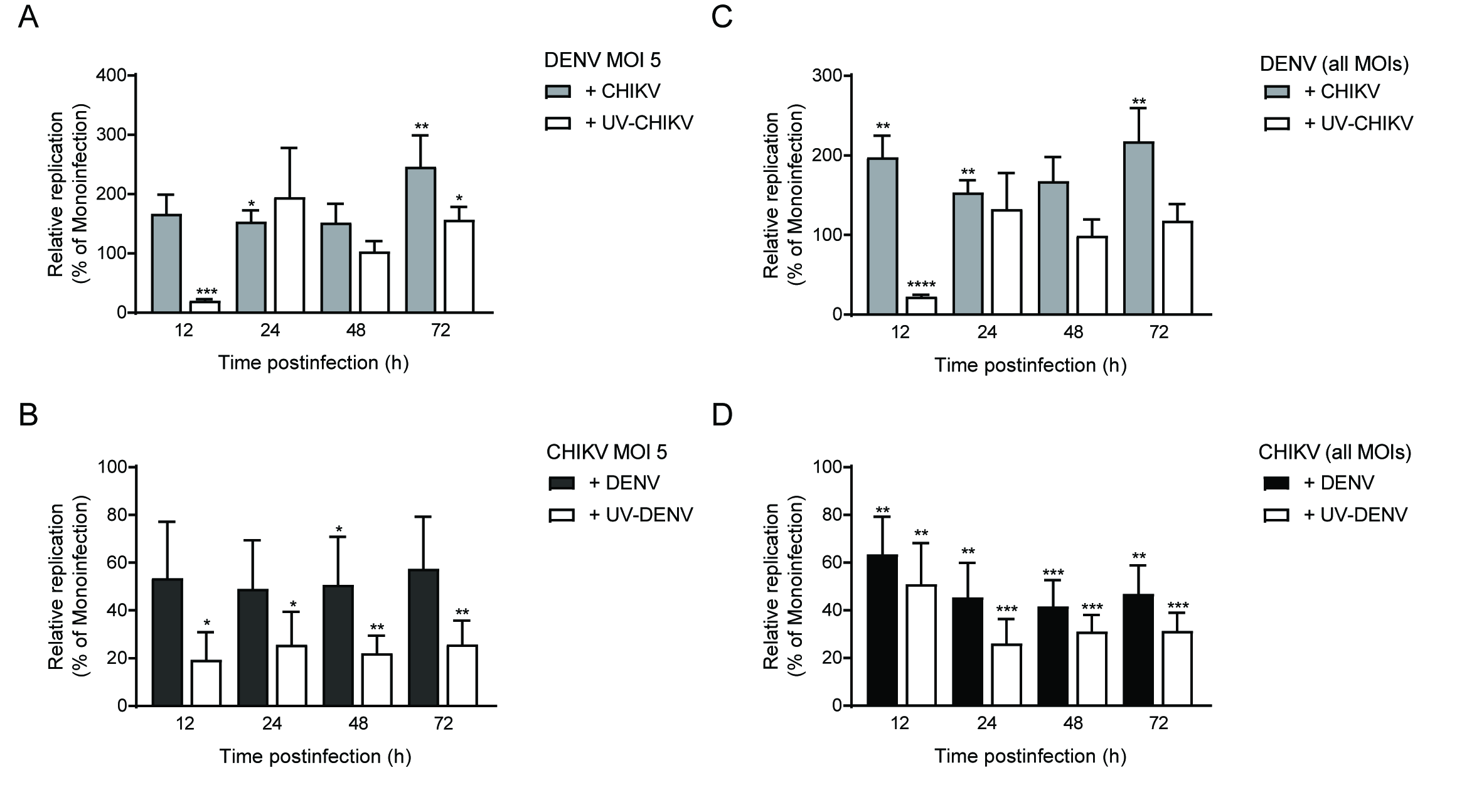

Supplement: S2 Fig — (A) Production of DENV MOI 5 during co-infection with CHIKV or UV-CHIKV relative to mono-infection; (B) Replication of CHIKV MOI 5 during co-infection with DENV or UV-DENV relative to CHIKV mono-infection; (C) Combined effects of co-infection (all MOIs) on DENV mono-infection; (D) Combined effects of co-infections on CHIKV mono-infections. Results are represented as mean ± SEM of three donors. (TIF) [file pntd.0005712.s002.tif]

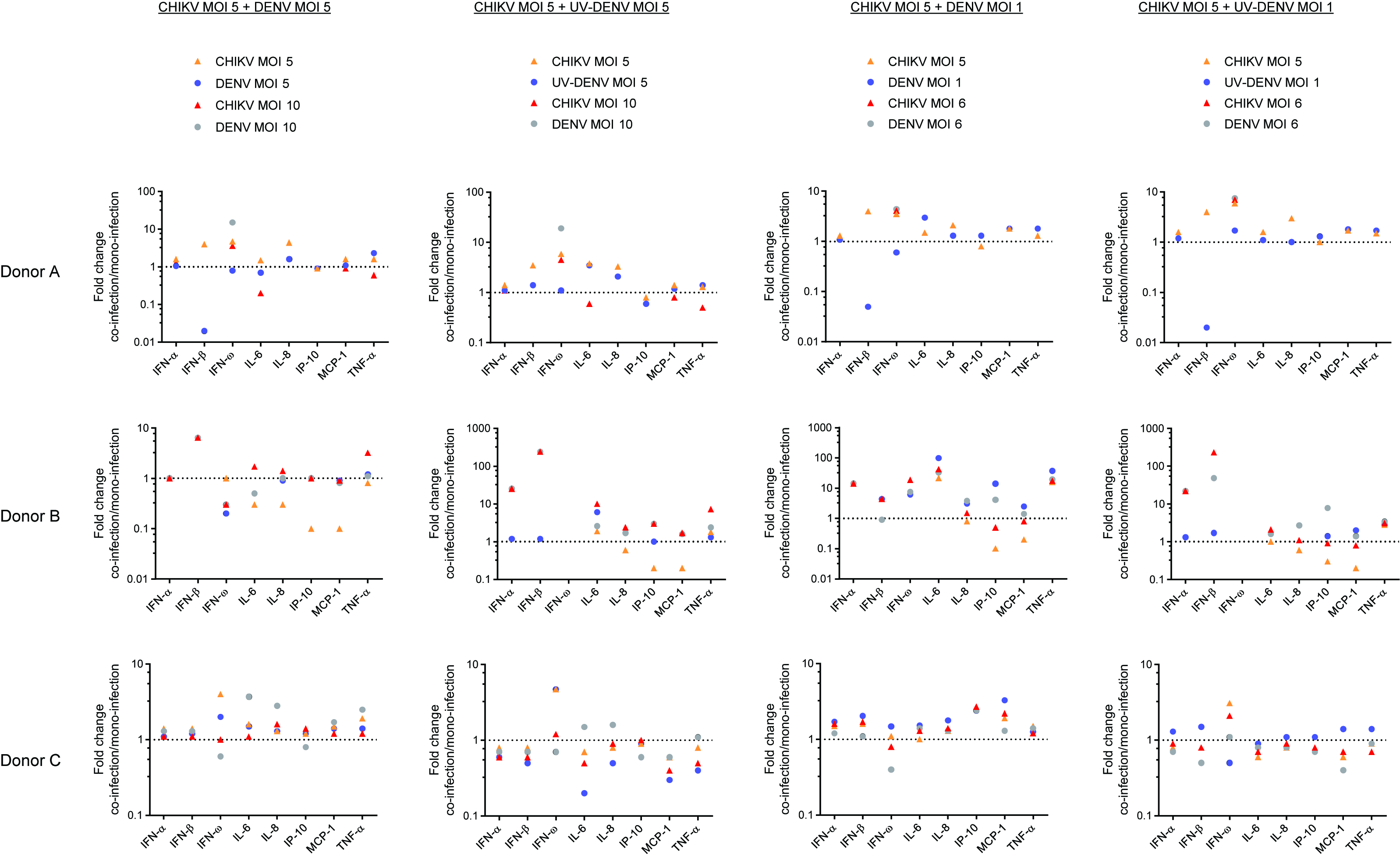

Supplement: S3 Fig — Each graph shows the fold-change of concentration of the immune factor during co-infection relative to the indicated mono-infection. All concentrations shown correspond to 24 hpi, except for that of IFN-ω, which was detectable on all donors at 48 hpi. (TIF) [file pntd.0005712.s003.tif]

## IFN- $\alpha$

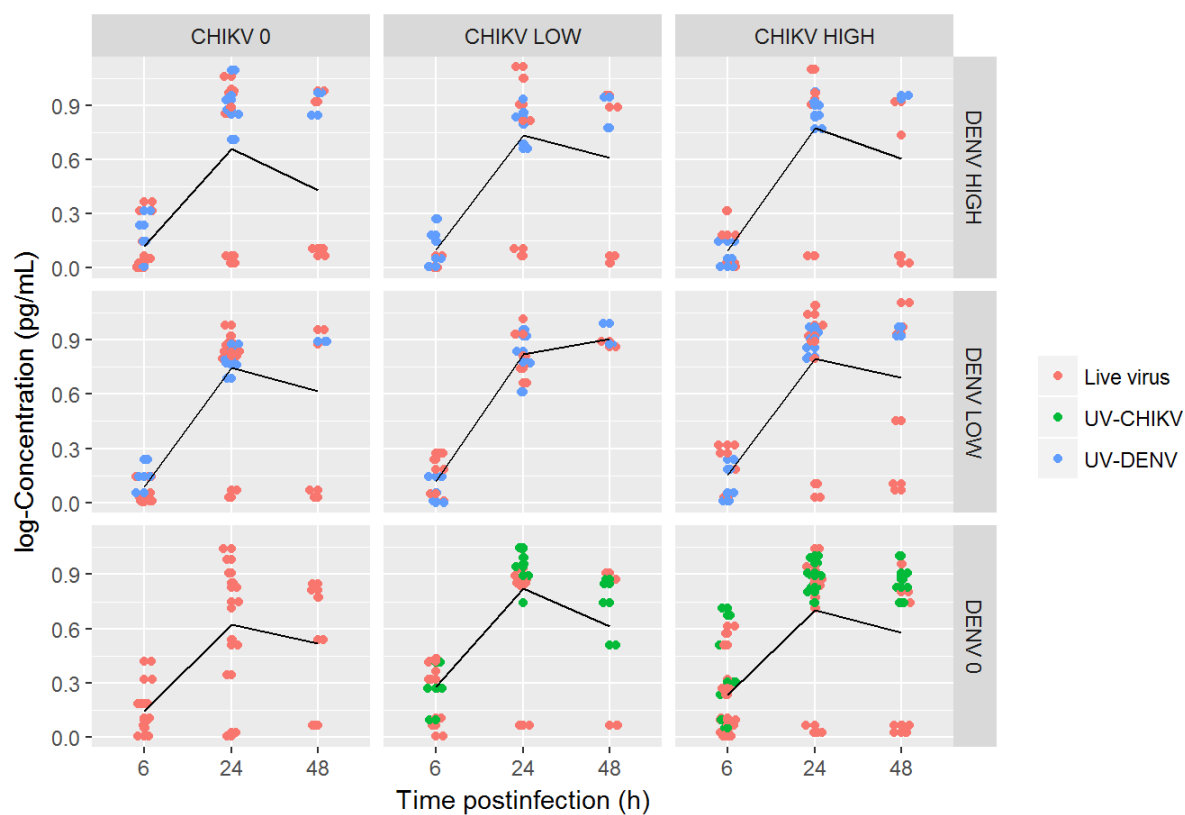

## IFN- $\beta$

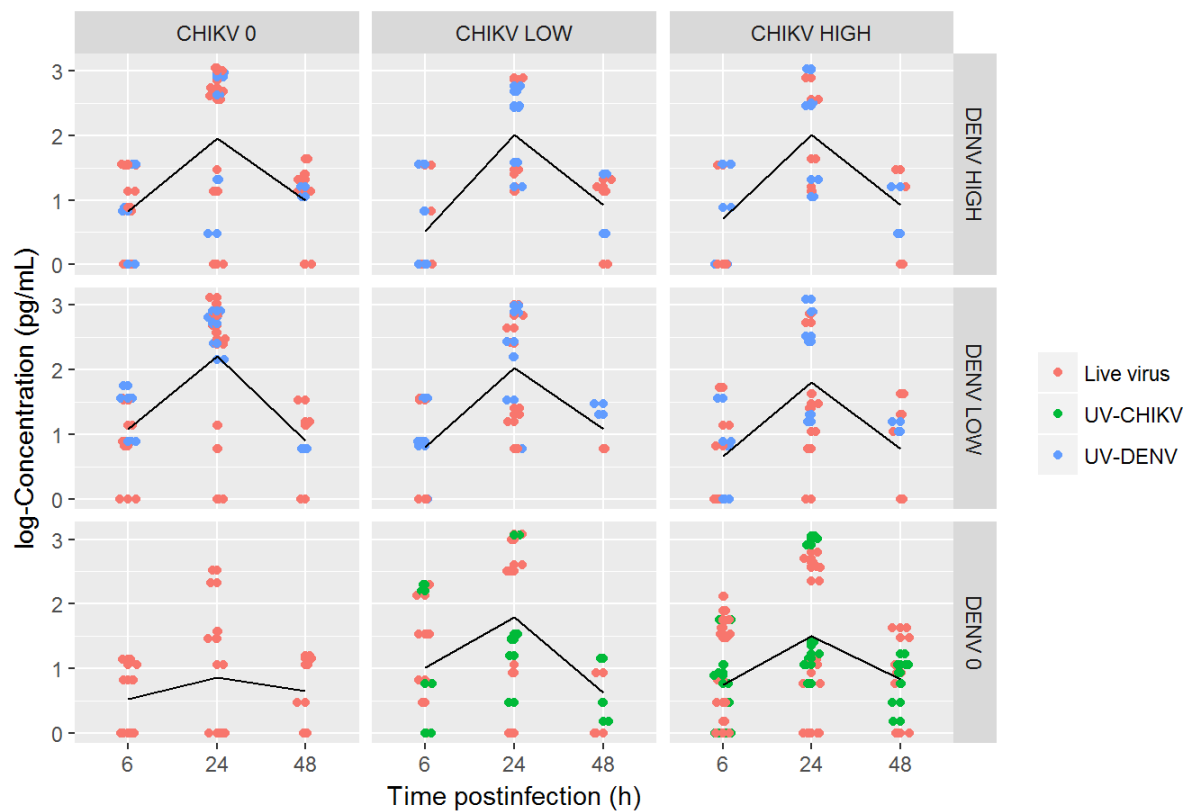

IFN- $\omega$

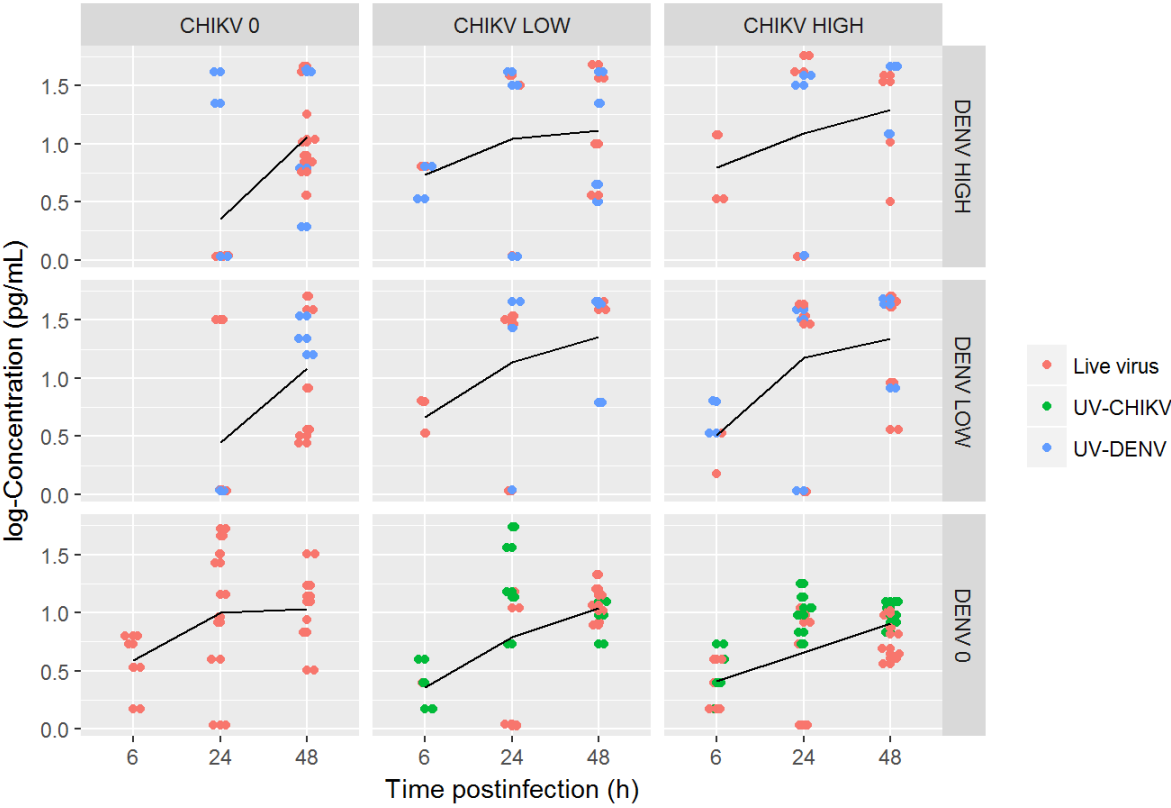

IL-6

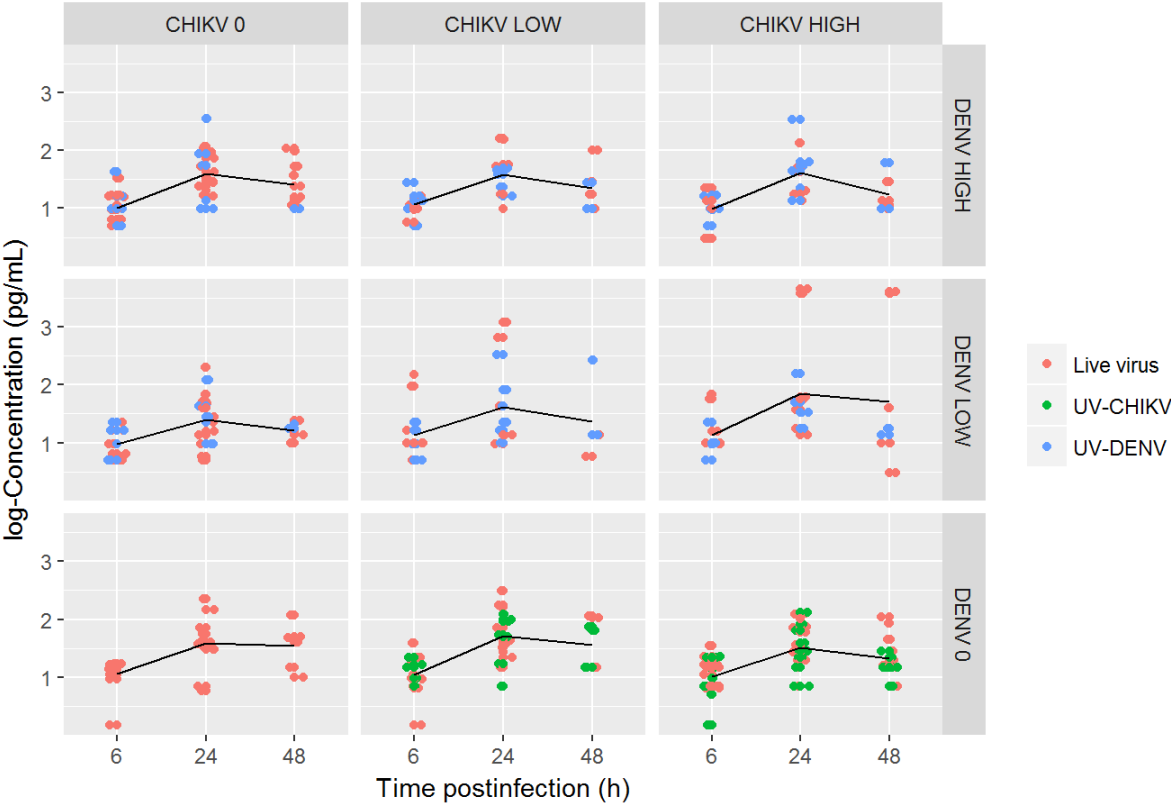

## IL-8

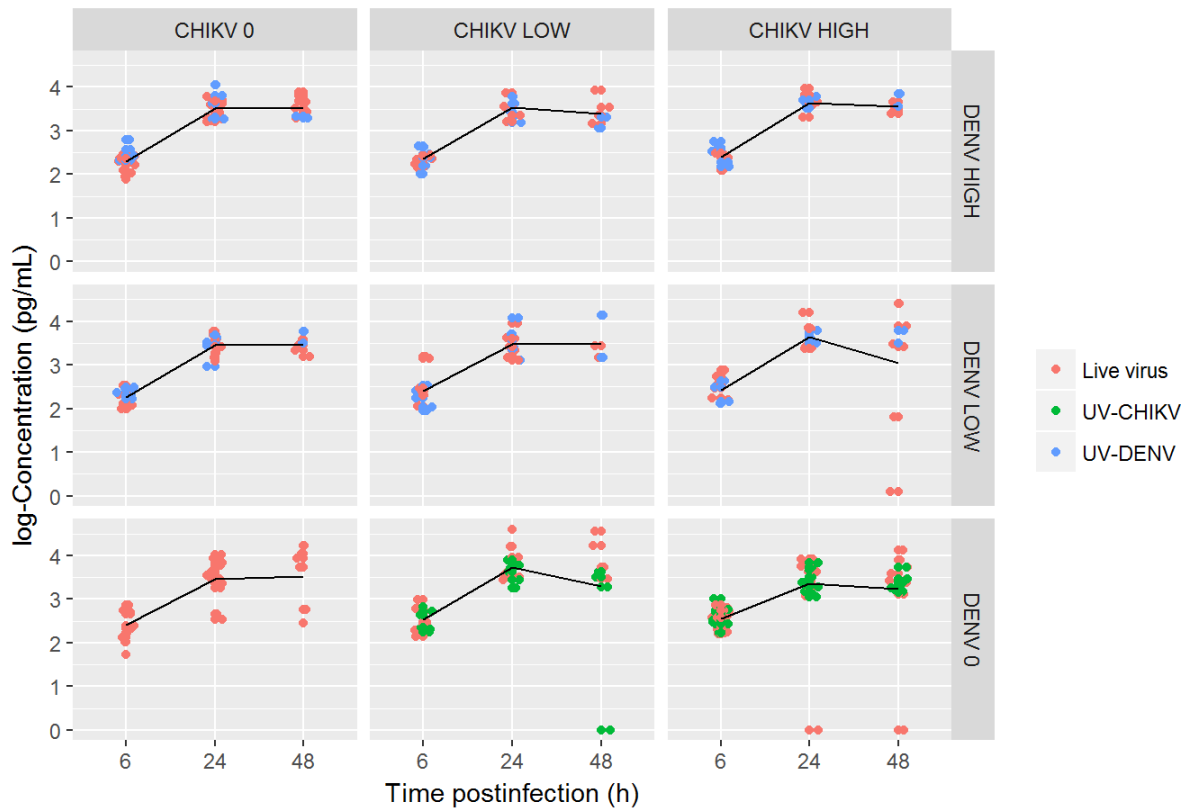

## IP-10

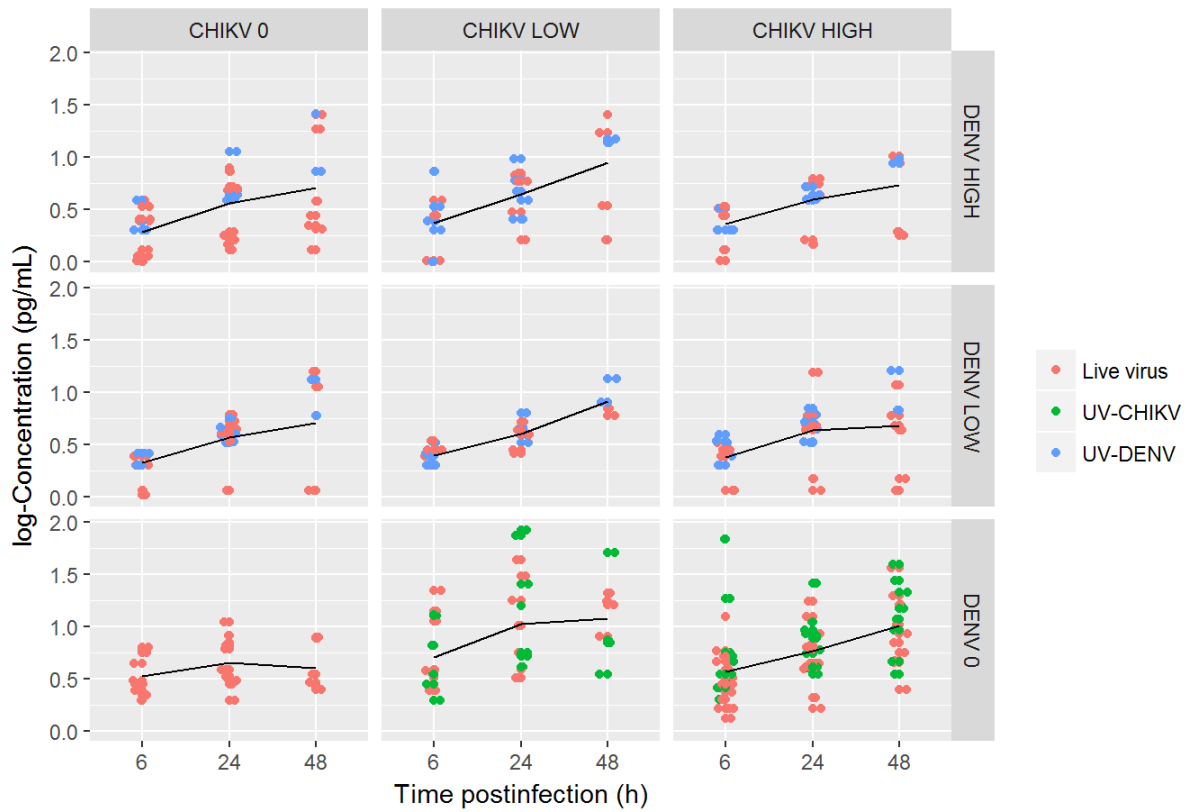

## MCP-1

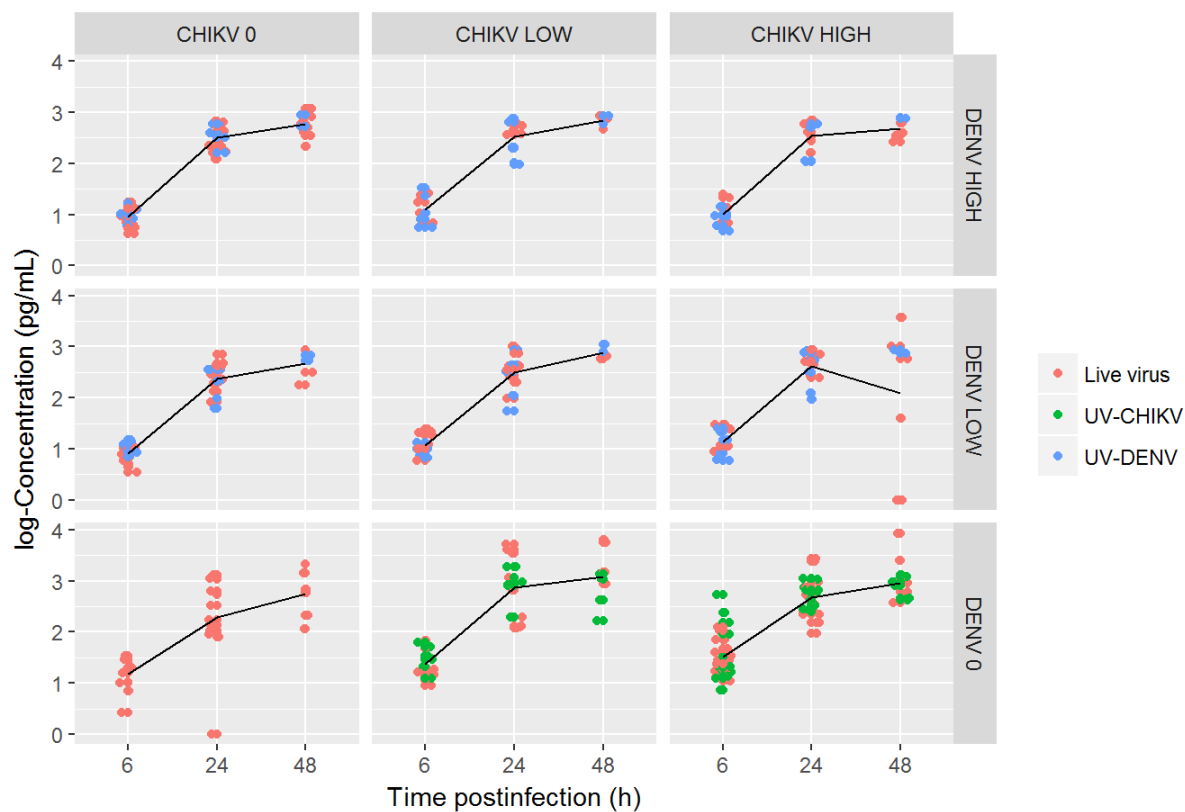

## TNF- $\alpha$

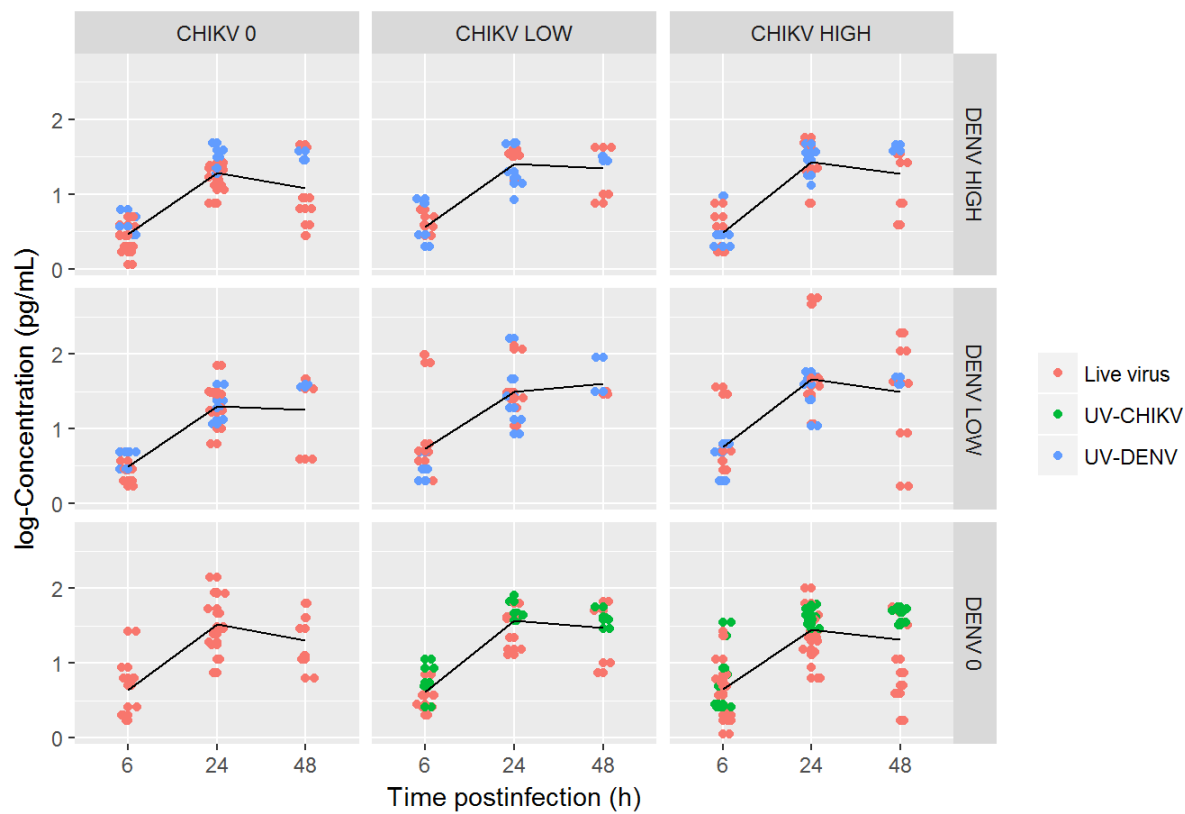

Supplement: S4 Fig — Each graph shows kinetics of the immune factor production during mono- and co-infections. High/ low represents results for MOI 1, 2 and MOI 5, 6 and MOI 10 respectively. All available data of 3 donors are shown. (PDF) [file pntd.0005712.s004.pdf]
